# Dexmedetomidine Protects Human Renal Tubular Epithelial HK-2 Cells against Hypoxia/Reoxygenation Injury by Inactivating Endoplasmic Reticulum Stress Pathway

**DOI:** 10.22074/cellj.2021.7220

**Published:** 2021-08-29

**Authors:** Mingyu Zhai, Mingming Han, Xiang Huang, Fang Kang, Chengwei Yang, Juan Li

**Affiliations:** Department of Anesthesiology, The First Affiliated Hospital of USTC, Division of Life Sciences and Medicine, University of Science and Technology of China, Hefei, Anhui, China

**Keywords:** Acute Renal Injury, Dexmedetomidine, Endoplasmic Reticulum Stress, Human Renal Tubular Epithelial

## Abstract

**Objective:**

The study was aimed to investigate the effects and potential mechanisms of Dexmedetomidine (Dex) on hypoxia/reoxygenation (H/R) injury in human renal tubular epithelial HK-2 cells.

**Materials and Methods:**

In this experimental study, HK-2 cells were divided into four groups: control group, Dex group, H/R group, and Dex+H/R group. The cells in control group received no treatment, and cells in Dex group were only treated with 0.1 nmol/L Dex. The cells in H/R group and Dex+H/R group were all treated with H/R (hypoxia for 24 hours and normoxia for 4 hours), and only the cells in Dex+H/R group were pre-administrated with 0.1 nmol/L Dex. Following treatments at 37˚C for 28 hours, cell viability and apoptosis were measured by MTT assay and flow cytometry, respectively. Also, the expressions of hypoxia-inducible factor 1 (HIF-1α), glucose-regulated protein 78 (GRP78), C/EBP homologous protein (CHOP), caspase-12 and cleaved caspase-3 were determined by western blot.

**Results:**

The cell viability was significant decreased in H/R group compared with control group (P<0.05), while was significantly increased in Dex+H/R group compared with that in H/R group (P<0.05). However, the change tendency of the cell apoptosis was opposite to that of cell viability. Compared with H/R group, the expression of HIF-1α was evidently up-regulated, while GRP78, CHOP, capase-12 and cleaved caspase-3 expressions were all obviously down- regulated in Dex+H/R group (P<0.05). In addition, the concentrations of malondialdehyde (MDA) in H/R group and Dex+H/R group were 1.68 ± 0.22 nmol/mgprot and 0.85 ± 0.16 nmol/mgprot, respectively. The superoxide dismutase (SOD) activity was higher in Dex+H/R group (121 ± 11 U/L), which which was more than twice larger than that in H/R group (57 ± 10 U/L).

**Conclusion:**

Dex could promote cell viability and inhibit apoptosis through up-regulating HIF-1α, reducing endoplasmic reticulum (ER) stress and mediating oxidative stress, thus ameliorating the H/R injury.

## Introduction

Acute renal injury (ARI) induced by ischemia/ reperfusion (I/R), and is a common event in trauma, hemorrhage and resuscitation. ARI may occur more frequently during several kinds of surgeries, including aortic surgery, cardiopulmonary bypass surgery and renal transplantation (1). Renal tubular epithelial cells, one of the main kidney parenchymal cells, are most susceptible to ischemic injury compared to other nephron segments. Tubular epithelial cells apoptosis is introduced as a main renal I/R injury feature. There is multiple evidence that anesthetics used during surgery show not only anesthetic action but also protective effects by reducing apoptosis during I/R (2, 3). 

Dexmedetomidine (Dex) is a α_2_ adrenergic receptor agonist, and showed a variety of effects, including sedation, anti-anxiety, analgesia, sympatholytic properties (4, 5). Dex is widely used in the operating room and intensive care units (ICUs). And recent animals and clinical studies showed reno-protective effects of Dex (6- 8). A study by Chen et al. (9) demonstrated that Dex could ameliorate diabetic hyperglycemia-exacerbated cerebral I/R injury via the suppression of inflammation, apoptosis and oxidative stress. Although, the effects and potential mechanisms of Dex on I/R injury in ARI remain to be further elucidated.

Previous studies have shown that cell apoptotic activation after I/R is driven by the loss of mitochondrial stability, thus contributing to the release of mitochondrial and cytochrome C (10-12). These events would result in mitochondrial dysfunction and caspase activation, contributing to the apoptosis after reperfusion. Qin et al. (11) indicated that Rhynchophylline could inhibit the apoptosis of myocardial I/R-induced cardiomyocytes by regulating the expression levels of caspase-3 and caspase-9. In addition, hypoxia inducible factor-1alpha (HIF-1α) plays an important role in maintaining oxygen homeostasis, regulating the expression of a series of hypoxia-related genes, and sensing and transmitting hypoxia signals (13). A recent study has shown that HIF-1α may ameliorate brain damage during I/R by reducing cell apoptosis (14). Post-treatment such as ischemia may be associated with up-regulating HIF-1α expression in the kidney of ischemia reperfusion rats, thus reducing the ischemia damage and hypoxia to the kidney (15). However, the relationships between cell apoptosis and renal protective effects of Dex, as well as HIF-1 α has remained unknown.

In addition, endoplasmic reticulum (ER), one of the largest organelle in eukaryotic cells, plays important roles in maintaining homeostasis. While cells need more ER function, over ER its capacity, this may lead to accumulating unfolded or misfolded proteins in the ER and alterations in the calcium homeostasis. , that called ER stress. Studies have shown that moderate ER stress is a kind of self-protection mechanism. These mechanisms, provided by activating unfolded protein reactions, temporarily inhibiting protein synthesis, and restore ER steady state to maintain cell survival (16, 17). However, excessive or prolonged ER stress may lead to cell apoptosis or necrosis, resulting in organ and tissue damage (18, 19). ER stress can be also triggered by various stimuli, such as ischemia, hypoxia, oxidative stress, glucose starvation, and elevated protein synthesis, which is one of the important ways to induce cell apoptosis(20, 21). There are studies that indicated the ER stress involvement in the reperfusion injury of vital organs such as heart, brain and kidney (22, 23). It is still unclear whether ER stress is involved in the kidney protection of Dex. 

This study was aimed to explore the effects and potential mechanisms of Dex on renal tubular epithelial cells, which were treated by hypoxia/reoxygenation (H/R). This allowed us to have a broader knowledge of Dex treatment in ARI.

## Materials and Methods

This research was performed after receiving the Ethics approval from the Ethics Communication of University of Science and Technology of China (2019-N(A)-243).

### Cell culture

In this experimental study, human renal tubular epithelial HK-2 cells were purchased from American Type Culture Collection (ATCC^®^ CRL-2190™, Manassas, VA, USA). The cells were maintained in Dulbecco’s modified Eagle medium/Nutrient Mixture F12 Ham (DMEM/F12, 3:1 Mixture, Thermo Fisher Scientific, Inc., Waltham, MA, USA) supplemented with 10% fetal bovine serum (FBS, Thermo Fisher Scientific, Inc., Waltham, MA, USA), 100 U/mL penicillin and 100 µg/mL streptomycin (Invitrogen, Thermo Fisher Scientific, Inc., Waltham, MA, USA) at 37˚C in a humidified atmosphere of 5% CO_2_ . The media was changed every 3-4 days, and the cells were passaged after reaching 80% confluence.

### Grouping and hypoxia/reoxygenation model establishment

Human renal tubular epithelial HK-2 cells were divided into 4 groups using a random number table control group, Dex group, H/R group, and Dex+H/R group. The HK-2 cells in control group were incubated at normoxia condition in 37˚C for 28 hours. For Dex group, different concentrations of Dex (0.01, 0.1, 1, and 10 nmol/L) or 0.1 nmol/L (Sigma-Aldrich, Merck KGaA, Darmstadt, Germany) was added to the culture medium with HK-2 cells. After incubated for 2 hours at 37˚C, the cells were transferred to the culture medium without Dex and incubated at normoxia condition for 28 hours in 37˚C. In the H/R group, cells, which were firstly incubated in an anaerobic chamber (Shanghai Lishen scientific instrument co. LTD, Shanghai, China) at 37˚C for 24 hours, then incubated at normoxia condition for 4 hours at 37˚C. In Dex+H/R group, after 2 hours incubation at 37˚C with different concentrations of Dex (0.01, 0.1, 1, and 10 nmol/L) plus culture medium , then, the cells were transferred to the culture medium, without Dex, under H/R group cells condition. Each experiment was repeated five times.

### \MTT (3-(4,5-dimethylthiazol-2-yl)-2,5-diphenyltetra-zolium bromide) assa

After treated with the different concentrations of Dex, the HK-2 cells were seeded into 96-well plates at a density of 5000 cells/well, and then MTT assay was used to measure the cell viability rate. Briefly, 100 µL fresh DMEM containing 1 mg/mL MTT (Sigma-Aldrich, Merck KGaA, Darmstadt, Germany) was utilized to replace 100 µL DMEM containing 10% FBS. After incubation at 37˚C for 4 hours，150 µL dimethyl sulfoxide was added to dissolve the formazan crystals. Then, the absorbance (optical density, OD) was detected at 568 nm using a microplate reader (Model ST-360, Thermo, Inc, MULTISKAN MK3, CA, USA). The whole experiment was performed in triplicate. The following cell survival ratio was employed:

R (%)=A/B * 100%

R: cell survival ratio, A: OD (experiment)–OD (blank), B: OD (control)–OD (blank).

### Apoptosis assay

HK-2 cells of logarithmic growth phase were diluted in DMEM medium containing 10% fetal bovine serum (FBS, Thermo Fisher Scientific, Inc., Waltham, MA, USA) to a final concentration of 5×10^5^ cells/mL. Cells were suspended in a concentration of 1×10^6^ cells and incubated for 24 hours. Then, the cells were digested with pancreatic enzyme (Beyotime Institute of Biotechnology, Shanghai, China). After 3 minutes, the cells were harvested, washed with phosphate buffer saline (PBS, Sinopharm Pharmaceutical Co. Ltd, Shanghai, China) and re-suspended in 1× kit binding buffer. Then, the ratio of apoptotic cells was examined using an Annexin V-fluorescein isothiocyanate/PI kit (BD PharMingen, San Diego, CA, USA). After, cells were incubated with Annexin V and PI according to the manufacturer’s instructions for 15 minuts at 26˚C in the dark, subjected to FACSCalibur flow cytometry analysis (BD Biosciences, San Jose, CA, USA), using Cell Quest Pro 5.2 software (BD Biosciences).

### Western blot analysis

Total protein was isolated using a Total Cell Protein Extraction kit (EMD Millipore, Billerica, MA, USA) based on the manufacturer’s instructions. The lysate was centrifuged at 15,000 rpm for 30 minutes to obtain the supernatant. Then, protein concentrations were determined using BCA Protein Assay kit (Thermo Fisher Scientific, Inc., Waltham, MA, USA). Subsequently, an equivalent amount of protein (30 µg/lane) from each sample was separated by 12% sodium dodecyl sulfate-polyacrylamide gel electrophoresis (SDS-PAGE, Sinopharm Group, Shanghai, China), and then transferred to polyvinylidene difluoride (PVDF, EMD Millipore, Billerica, MA, USA) membranes (Sigma-Aldrich, Merck KGaA, Germany). After blocking in 5% non-fat milk at room temperature (approximate 26˚C) for 2 hours, the membranes were incubated with primary antibodies at 4˚C overnight as follows: rabbit anti-hypoxia inducible factor-1alpha (HIF-1a) antibody (1:1000; Abcam, UK), rabbit anti-glucose regulation protein 78 (GRP78) antibody (1:1000; Abcam, UK), rabbit anti-human C/EBP homologous protein (CHOP) antibody (1:2000; Abcam, UK) and rabbit anti-activation of the caspase-12 and caspase-3 one reactance antibody (1:1000; Abcam, UK). The β-actin antibody (1:200; Abcam, UK) was served as the loading control. After washing incubated membranes with Tris-buffered saline with 0.1% Tween-20 three times, membranes incubated with goat anti-rabbit IgG H&L labelled with horseradish peroxidase (1:1000; Jackson ImmunoResearch Laboratories, Inc., USA) for 1 hour. Finally, the protein bands were visualized with an enhanced chemiluminescence detection kit (Santa Cruz Biotechnology Inc, CA, USA), and quantified by densitometric analysis using Quantity One software (National Institutes of Health, Bethesda, MD, USA). 

### Measurement of malondialdehyde concentration and superoxide dismutase activity

Five petri dishes were taken from each group, and the medium was discarded. Afterwards, the cells were digested by trypsin and re-suspended with PBS. Using ultrasonic cell disruptor (Bilon-150y, Shanghai Bilang instrument co., LTD., Shanghai, China), cells were lysed Evaluating the extent of oxidative stress in H/R and Dex treated HK-2 cells, the concentration of malondialdehyde (MDA) and superoxide dismutase (SOD) activity in cells were measured using assay kits for MDA (A003- 1) and total SOD (A001-1) from Nanjing Jiancheng Bioengineering Institute (Nanjing, China), according to the manufacturer’s instructions.

### Statistical analysis

Data are expressed as mean ± standard deviation (SD). Statistical differences between groups were determined using one-way analysis of variance followed by a Turkey’s post hoc test. All statistical analysis was performed using SPSS 13.0 (SPSS Inc., Chicago, IL, USA), and P<0.05 was considered to be statistically significant.

## Results

### The dexmedetomidine effects on HK-2 cells viability and apoptotic rate 

**Fig.1:**
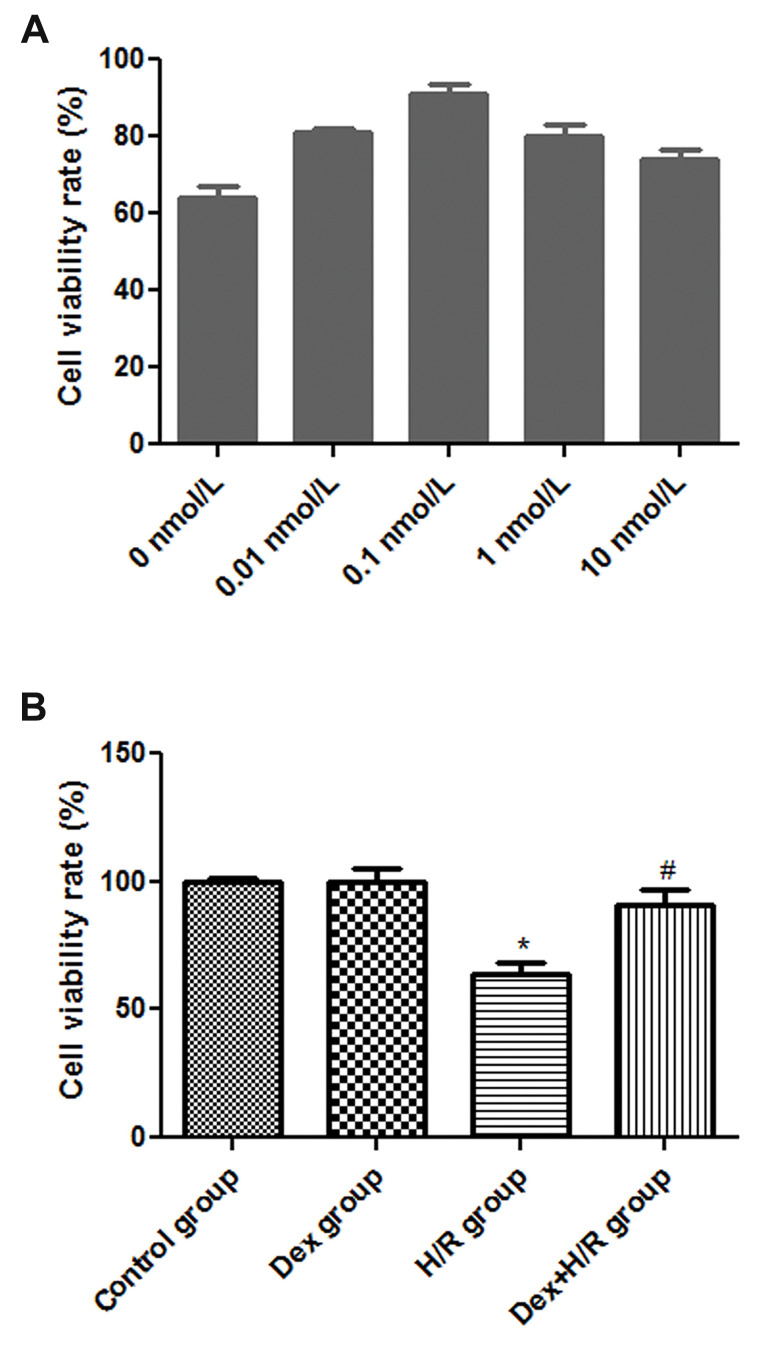
The cell viability of HK-2 cells with different treatments was measured by MTT assay. **A.** The cell viability rates of HK-2 cells were determined after treated with different concentrations of Dex. **B. **The cell viability rates were measured in different groups. Dex; Dexmedetomidine, H/R; Hypoxia/reoxygenation, *; P<0.05, compared with control group, and ^#^ ; P<0.05, compared with H/R group

When the concentration of Dex was 0.1 nmol/L, the cell viability rate of H/R-induced HK-2 cells showed higher than that treated with other concentrations of Dex (Fig .1A). Therefore, 0.1 nmol/L Dex was selected for subsequent experiment. The cell viability rate in H/R group (64 ± 4.51%) was significantly decreased in comparison with control group (100 ± 1.3%, P<0.05, Fig .1B). Administration of 0.1 noml/L Dex, resulted insignificant increase of cell viability in the Dex+H/R group (91 ± 6.13%), near the control group level (100 ± 1.3%, P>0.05, Fig .1B). 

**Fig.2:**
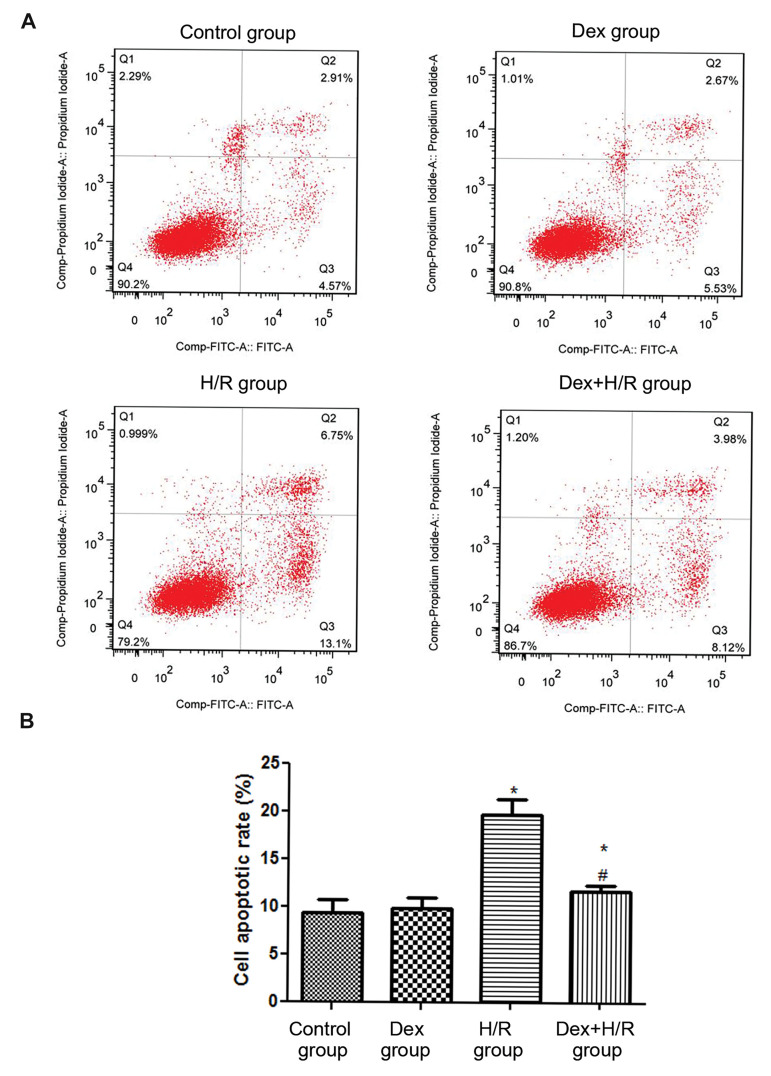
Cell apoptotic rate comparison between all groups was performed by apoptosis assay. **A. **Flow cytometric images and **B.** The cell apoptotic rates in different groups. Dex; Dexmedetomidine, H/R; Hypoxia/reoxygenation, * ; P<0.05, compared with control group, and ^#^ ; P<0.05, compared with H/R group.

The change in the rate of apoptosis was in contrast with rate of viability (Fig .2). Compared with the control group (9.42 ± 1.31%), the cell apoptotic rate in H/R group (19.78 ± 1.56%) was significantly increased (P<0.05, Fig .2B). However, after 0.1 nmol/L Dex treatment, the cell apoptotic rate was 11.79 ± 0.58%, which showed that Dex could significantly inhibit cell apoptosis induced by H/R (Fig .2B). 

### The effects of dexmedetomidine on the expressions of HIF-1α and apoptosis-related proteins 

**Fig.3:**
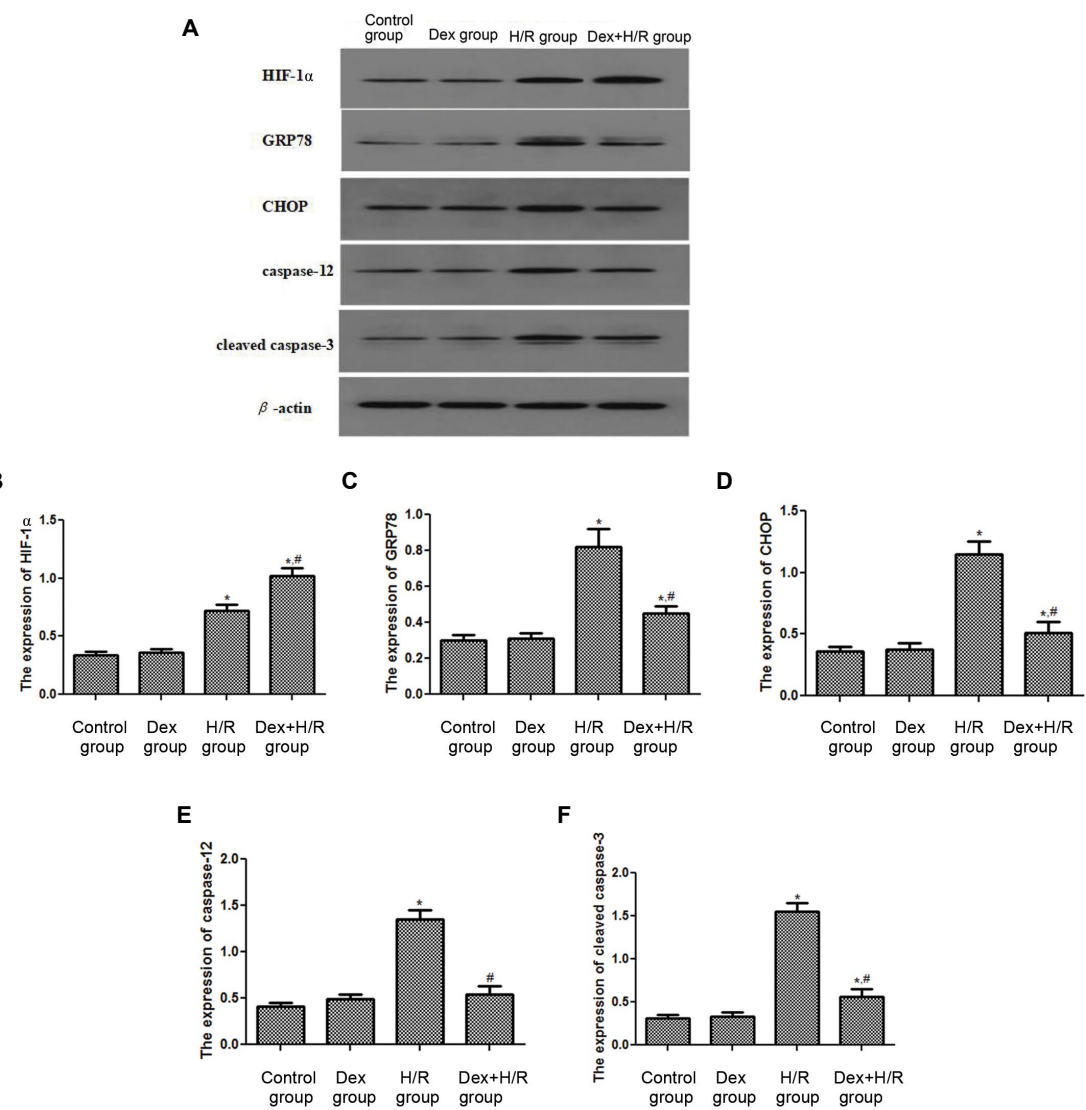
The effects of Dex on the expressions of HIF-1α, GRP78, CHOP, caspase-12 and caspase-3 were evaluated by western blot. **A.** The expression levels of HIF-1α, GRP78, CHOP, caspase-12 and cleaved caspase-3 were assessed by western blot analysis. The expression levels of **B. **HIF-1α, **C. **GRP78, **D. **CHOP, **E.** caspase-12, and **F.** The cleaved caspase-3 by gray analysis of western blot. Dex; Dexmedetomidine, H/R; Hypoxia/reoxygenation, * ; P<0.05, compared with control group, and ^#^ ; P< 0.05, compared with H/R group.

By the western blot of HIF-1α, the expression level of HIF-1α was greatly up-regulated in H/R group compared with control group (P<0.05). Also, after Dex treatment, the HIF-1α expression was increased compared with H/R group (P<0.05, Fig .3A, B). The results of apoptosis-related proteins were shown in Figure 3A, C-F. Compared with control group, the expression levels of GRP78, CHOP, caspase-12, and cleaved caspase-3 were all significantly increased in H/R group (P<0.05). In Dex+H/R group, the levels of GRP78, CHOP, caspase-12, and cleaved caspase-3 were all lower than those in H/R group (all P<0.05). In addition, after treated with Dex, the expression of caspase-12 restored to the same level of control group (P>0.05). 

### The effects of dexmedetomidine on the concentration of malondialdehyde and superoxide dismutase activity

**Fig.4:**
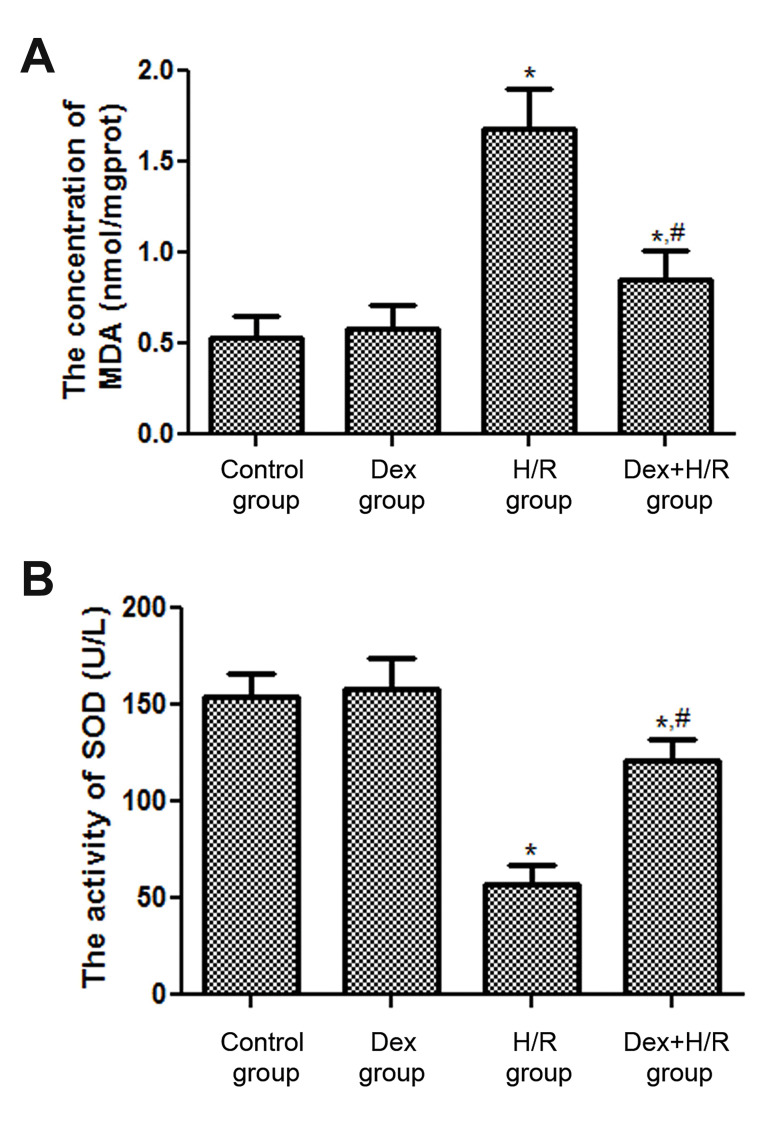
The effects of Dex on the MDA content and SOD activity. The concentrations of **A.** MDA and the activity of **B. **SOD. Dex; Dexmedetomidine, MDA; Malondialdehyde, SOD; Superoxide dismutase, H/R; Hypoxia/reoxygenation, *; P<0.05, compared with the control group, and ^#^ ; P<0.05, compared with H/R group.

In order to understand the mechanism of Dex relieving H/R damage, two oxidative stress related markers were identified in this study. The concentration of MDA in H/R group (1.68 ± 0.22 nmol/mgprot) was obviously higher than that in control group (0.53 ± 0.12 nmol/ mgprot, P<0.05, Fig .4A). The concentrations of MDA in Dex+H/R group and H/R group were 0.85 ± 0.16 nmol/mgprot and 1.68 ± 0.22 nmol/mgprot, which showed a 49.4% decrease in Dex+H/R group (Fig .4A). The trend of SOD activity was opposite to the concentration of MDA. Compared with control group, the SOD activity was significantly decreased in H/R group (P<0.05, Fig .4B). In addition, after Dex treatment, the SOD activity was rose to 121 ± 11 U/L, which was more than twice larger than that in H/R group (57 ± 10 U/L, Fig .4B).

## Discussion

ARI, a rapid decline in renal function, is caused by a variety of etiologies, that may influence people’s health and life (24). Dex, a kind of sedatives, has been reported to have the protective effect on renal injury (25). In this study, the effects and potential mechanisms of Dex on H/R injury in human renal tubular epithelial HK-2 cells were explored. The results showed that after 24 hours hypoxia and 4 hours reoxygenation, HK-2 cell viability rate was significantly decreased, while cell apoptotic rate was increased compared to control group. After Dex treatment, cell viability rate and apoptotic rate were restored to some extent. Dex could up-regulate the expression HIF-1α and inhibit the expressions of GRP78, CHOP, caspase-12, and cleaved caspase-3 in Dex+H/R group compared with the H/R group. In addition, Dex could improve H/R injury by decreasing the MDA content and enhancing SOD activity. All results indicated that Dex may have a protective effect on HK-2 cells after H/R injury.

In our study, Dex could increase the cell viability and inhibit the cell apoptosis after H/R injury. Several studies have indicated that HIF-1α plays an important role in the regulation of hypoxia-induced apoptosis and promotes cell survival by mediating cell adaptation to hypoxia (26). In this research, HIF-1α protein expression was up-regulated after H/R in HK-2 cells, indicating that HIF-1α was activated as an endogenous protective factor after H/R. A further increase in the HIF-1α expression after Dex administration, suggesting that the Dex protective role in improving H/R injury in human renal tubular epithelial cells may be related to the up-regulation of HIF-1α expression. A study of Zhang et al. (27) has reported that berberine protected renal tubular epithelial cells from hypoxia/high glucose-induced apoptosis by activating HIF-1α expression. Therefore, we speculated that Dex played a protective role in H/R injury by up-regulation of HIF-1α and suppressing cell apoptosis. However, the specific mechanism of up-regulation of HIF-1α by Dex remains to be further investigated.

In addition, studies have revealed that ER is a major contributor to cellular apoptosis and post-hypoxia injury (28). Our study showed that incubation of HK-2 cells in hypoxia condition increased the expression of GRP78, CHOP, caspase-12 and cleaved caspase-3. After Dex administration, this expression were down-regulated compared with H/R group. GRP78 is a partner protein of the ER, and is one of the classical markers for the stress of ER. Furthermore, GRP78 can promote proper folding of proteins as misfolded states appear as traps in the mesh cavity (29). When the ER stress occurs，the intracellular non-foldable protein response pathway is activated (30), which stimulates the transcription and synthesis of cell apoptotic marker protein, CHOP (31). CHOP is the specific transcription factor of ER stress. Oyadomari and Mori (32) reported that the CHOP gene knockout could enhance the cells resistance to ER stress induced apoptosis, and suggested that CHOP plays an important role of promoting apoptosis in the ER stress. In addition, another study has shown that the CHOP expression would increase substantially when severe ER stress occurs, and eventually induce cell apoptosis (33). Caspase family is a cell apoptosis mediator. Zhao et al. (34) showed that LipoxinA4 could protect myocardial I/R injury via a mechanism related to down-regulation of caspase-12 and inhibition of apoptosis. Cleaved caspase-3 is produced after caspase-3 shearing and is considered as a sign of apoptosis. Damarla et al. (35) demonstrated that the cleaved caspase-3 was activated in acute lung injury induced by lipopolysaccharide. Combined with our results, ER stress was overexpressed during apoptosis of renal tubular epithelial cells, and Dex may protect the renal by inhibiting excessive ER stress response, so as to reduce apoptosis of renal tubular epithelial cells caused by H/R.

It has been reported that oxidative stress mediated by reactive oxygen species (ROS) is involved in the pathogenesis of I/R injury (36). Oxygen free radical, one of the important renal I/R injury inducing factors, can produce MDA during the lipid peroxidation of unsaturated fatty acid (37). The concentration of MDA can often directly reflect the degree of lipid peroxidation in the body, and indirectly reflect the severity of free radicals attacks on cells(38). SOD, an antioxidant enzyme that scavenges oxygen free radical, plays an important role in protecting cells from oxidative damage (39). In addition, oxygen free radicals also can participate in cell signal transduction and apoptosis regulation (40). In our study, compared with H/R group, the MDA concentration in Dex+H/R groups was decreased, while the SOD activity was increased. These results indicated that Dex could regulate the oxidative stress reaction by decreasing MDA content and promoting SOD activity, and further to alleviate ARI induced by H/R. 

However, there are some limitations in this study. The therapeutic effects of Dex should be confirmed by animal model and* in vivo* studies. The relationship between the effects of Dex on the related genes (please insert genes name or refer them) expression and H/R injury improvement needs to be further study, which may be provided by gain and loss of function tests. Additionally, the mechanism of Dex on regeneration of human renal tubular epithelial HK-2 cells in H/R injury should be further investigated by scratching assay and epithelial-mesenchymal transition (EMT) evaluation.

## Conclusion

Here, we demonstrated that Dex can protect ARI by inhibiting cell apoptosis, excessive ERS response and regulating oxidative stress reaction. These results provide a theoretical basis for the possibility that Dex may be a potentially effective treatment strategy for patients undergoing kidney surgery
